# Direct-to-Consumer Teledermatology for Male Androgenetic Alopecia: Narrative Review

**DOI:** 10.2196/72704

**Published:** 2025-12-19

**Authors:** Finn Abeck, Inga Hansen-Abeck, Julian Kött, Edward Garrahy, Stefan W Schneider, Johannes von Büren

**Affiliations:** 1 Department of Dermatology and Venereology, University Medical Center Hamburg-Eppendorf, Martinistrasse 52, Hamburg, 20251, Germany, 49 15222837104; 2 Wellster Healthtech Group, Munich, Germany

**Keywords:** direct-to-consumer, teledermatology, telemedicine, androgenetic alopecia, male pattern hair loss, finasteride

## Abstract

This narrative review examines the advantages and disadvantages of direct-to-consumer teledermatology for the treatment of male androgenetic alopecia, finding that this treatment modality improves access to care, ensures high adherence rates, and enhances patient satisfaction, while raising concerns about increased drug costs.

## Introduction

Androgenetic alopecia (AA) is the most common form of hair loss [1]. Oral finasteride is the most effective pharmacological treatment for AA; however, its prescription-only status may limit accessibility [1]. Recently, direct-to-consumer (DTC) teledermatology platforms have gained popularity as a convenient way for patients to receive treatment without a traditional clinical outpatient appointment [2,3]. This narrative review examines the benefits and potential drawbacks of DTC treatment for AA.

## Methods

A narrative review was conducted on PubMed (February 2025) using the following keywords: telehealth, teledermatology, telemedicine, direct to consumer, digital health, telemedical care, androgenetic alopecia, male pattern hair loss, finasteride. In total, 16 records were identified. After screening and assessing eligibility, 8 records were excluded (records not addressing DTC AA treatment or study was not primary literature). Table 1 summarizes the characteristics of the included studies. Given the nature of narrative reviews, selection bias cannot be entirely ruled out.

**Table 1.** Studies reporting on the use of direct-to-consumer teledermatology for androgenetic alopecia treatment.

Study	Population/sample	Results	Conclusion
Abeck et al [2], 2022 (Germany)	2904 patients (oral finasteride)	55.4% had never consulted a physician due to AA^a^ 76.1% had not previously received treatment with oral finasteride Treatment barriers: low disease burden, long waiting times	DTC^b^ treatment for male AA has the potential to reach a significant number of untreated patients. Most patients requested repeat prescriptions, rated the medical care quality as comparable to in-person visits, and expressed a willingness to continue telemedicine care, all suggesting patient benefit.
Young et al [3], 2023 (United States)	8983 patients (oral finasteride or topical minoxidil)	81% reported hair regrowth or cessation of hair loss 91% never or rarely missed their medication 32% had never considered treating their hair loss before learning about the DTC platform	A DTC platform for AA treatment can increase access to care for a large patient population while ensuring high treatment adherence and achieving favorable outcomes for patients.
Abeck et al [4], 2023 (Germany)	2904 patients (oral finasteride)	Tremendous increase in the number of visits to DTC platforms for AA Most common reasons indicated for using the platform: convenience (79.1%) and discretion (32.8)	The significant increase in visits to DTC platforms for AA indicates a shift in medical care.
von Büren et al [5], 2023 (Germany)	2269 patients (oral finasteride)	79% reported positive changes in hair appearance 59% reported an improvement in self-esteem Full treatment adherence was reported in 87%	DTC teledermatology has the potential to enhance hair appearance and boost self-esteem. It can be an effective and safe treatment option for men with AA, though treatment-related adverse events should be carefully monitored during follow-up.
Abeck et al [6], 2024 (Germany)	1545 patients (topical gel of finasteride admixed with minoxidil)	62.2% reported positive changes in hair appearance 44.1% reported an improvement in self-esteem after 6 weeks Full treatment adherence was reported in 74.4%	According to patient-reported outcomes, topical finasteride/minoxidil appears effective and well-tolerated, though not superior to oral finasteride. Lower adherence to topical treatment should be considered when evaluating treatment options.
Jean-Pierre and Williams [7], 2024 (United States)	4 DTC companies	DTC companies with a 1.6-fold increase for oral finasteride compared to traditional pharmacies	Balancing the convenience of DTC treatment options with patient safety and quality of care is essential.
Buxo and Fakhoury [8], 2025 (United States)	5 DTC companies	3-month package: cheapest finasteride price from a DTC platform was 1.5 times the price of the cheapest wholesale pharmacies 12-month package: cheapest finasteride price from a DTC platform was 2.3 times the price of the cheapest wholesale pharmacies	Finasteride is considerably more expensive from DTC pharmacies compared to wholesale pharmacies.

a AA: androgenetic alopecia.

b DTC: direct-to-consumer.

## Results

### Overcoming Barriers to Hair Loss Treatment via DTC Teledermatology

DTC AA treatment offers the potential to improve access to care for patients who would otherwise potentially forgo treatment. Abeck et al [2] found that 55.4% of patients had never consulted a physician due to AA and 76.1% of patients had not previously received treatment with oral finasteride before undergoing telemedicine care. Treatment barriers included low disease burden, prolonged outpatient appointment waiting times, and shame [2]. A further study confirmed convenience and discretion as key motivators involved in choosing DTC treatment [4]. According to Young et al [3], almost one in three new patients had not considered hair loss treatment until they became aware of telemedical platforms.

### High Treatment Success and Patient Satisfaction With DTC Therapy

After 6 months, 81% of patients from a US platform reported hair regrowth or cessation of hair loss [3]. Similar results were reported among patients taking oral finasteride from a German platform: 79% reported positive changes in hair appearance and 59% reported an improvement in self-esteem after 6 weeks [5]. Slightly lower treatment success was reported by patients undergoing topical finasteride/minoxidil treatment [6]. In the study from Abeck et al [2] nearly all patients (97.7%) wished to continue therapy on the DTC platform, and most (81%) patients rated medical care via the platform as at least as good as, or better than, care provided in a previous outpatient appointment with a physician.

### Safety and Side Effects of DTC Finasteride Treatment

Adverse events (AEs) associated with DTC teledermatology treatment for AA were reported in 5%‐12% of patients, with most being mild [5,6]. DTC platforms can improve patient safety by implementing automated systems that prevent the simultaneous ordering of contraindicated medications. Additionally, DTC platforms can expand scientific knowledge through real-world digital data collection. Sexual AEs, such as decreased libido and erectile dysfunction, may be associated with finasteride usage [1]. The study by Abeck et al [6] using data from a German DTC platform found no difference in the incidence of sexual AEs between patients treated with topical finasteride and those treated with oral finasteride. In total, 2.3%‐2.5% of patients reported sexual AEs. This real-world data expands upon our knowledge of the incidence of potential AEs.

### Strong Adherence Rates in DTC Finasteride Treatment

Full treatment adherence with DTC teledermatology treatment for AA varied from 74.4% (topical finasteride/minoxidil) to 87% (oral finasteride) in patients visiting a German platform [5,6]. Additionally, 91% of patients from a platform in the United States reported never or rarely missing their medication [3].

### Comparing the Cost of DTC versus Traditional AA Treatment

Buxo and Fakhoury [8] compared the prices of generic finasteride in 5 DTC platforms and 3 wholesale pharmacies in the United States. The study identified that finasteride was significantly more expensive when purchased from DTC pharmacies. Specifically, the lowest 3-month price from DTC pharmacies was about 1.5 times higher than that from the cheapest wholesale pharmacies. Similar price differences were described by Jean-Pierre and Williams [7], as DTC companies showed a 1.6-fold increase for oral finasteride compared to traditional pharmacies. However, indirect costs of doctor visits, such as transport costs and lost working time, must also be accounted for when assessing prices [8].

## Discussion

DTC platforms for male AA treatment have the potential to improve access to care while offering treatment success and practical benefits. The high adherence rate to teledermatology, together with treatment-related AE rates comparable to those of conventional care [9], justifies low-threshold access. However, continuous monitoring of potential AEs like sexual dysfunction associated with finasteride remains essential. Higher drug prices are a drawback of DTC care. Studies on the long-term care and safety of patients from DTC platforms are necessary to further assess the benefits of telemedical care for male AA.

## Abbreviations

AA: androgenetic alopecia
AE: adverse event
DTC: direct-to-consumer
